# Associations of Physical Activity and Dietary Habits with Migraine Frequency and Intensity in Adults: A Cross-Sectional Study

**DOI:** 10.3390/medicina62050837

**Published:** 2026-04-28

**Authors:** Ardiana Murtezani, Deart Jakupi, Shkurta Rrecaj Malaj, Vjosana Qeriqi, Zana Ibraimi

**Affiliations:** 1 Department of Physical Medicine and Rehabilitation, Faculty of Medicine, University of Prishtina, 10000 Prishtina, Kosovo; ardiana.murtezani@uni-pr.edu (A.M.); shkurta.malaj@uni-pr.edu (S.R.M.); 2 Physical Medicine and Rehabilitation Clinic, University Clinical Center of Kosova, 10000 Prishtina, Kosovo; 3 Department of Pharmacy, Faculty of Medicine, University of Prishtina, 10000 Prishtina, Kosovovjosana.qeriqi@uni-pr.edu (V.Q.)

**Keywords:** headache disorders, perceived stress, body mass index, sleep duration, fast food consumption, aura

## Abstract

*Background and Objectives*: Migraine is a common neurological condition that can affect daily life and well-being. Lifestyle factors such as physical activity, sleep, diet, and stress are often discussed in relation to migraine, but their role is not always consistent. This study aimed to examine how selected lifestyle factors are related to migraine frequency and intensity in adults, with a focus on physical activity and dietary habits. *Materials and Methods*: A cross-sectional study was conducted among 300 adults recruited through an online migraine-focused community from 1 January to 28 February 2026. Participants completed a questionnaire addressing migraine history, frequency and duration of attacks, pain intensity, physical activity, sleep duration, perceived stress, and dietary habits. Associations were assessed using Spearman correlation and multiple linear regression analyses. *Results*: Most participants were female (88%), and 48% reported physician-diagnosed migraine. Mean migraine intensity was 7.44 ± 1.72. Migraine intensity was positively correlated with migraine frequency (rs = 0.31, *p* < 0.001), episode duration (rs = 0.48, *p* < 0.001), perceived stress (rs = 0.17, *p* < 0.05), and sleep duration (rs = 0.16, *p* < 0.05). Migraine frequency was correlated with fast food consumption (rs = 0.23, *p* < 0.01) and BMI (rs = 0.25, *p* < 0.01). In regression analysis, migraine frequency (β = 0.17, *p* = 0.005), perceived stress (β = 0.23, *p* = 0.006), and aura (β = −0.19, *p* = 0.033) were significant predictors of migraine intensity. Physical activity was not significantly associated with migraine intensity or frequency. *Conclusions*: Migraine intensity was most consistently related to perceived stress and migraine frequency, whereas migraine frequency was associated with dietary factors and BMI. Physical activity was not associated with migraine outcomes. These findings suggest that lifestyle factors are related to migraine characteristics, although the cross-sectional design does not allow conclusions about causality.

## 1. Introduction

Migraine is one of the most common neurological conditions and remains a major source of disability worldwide [1]. It affects people across different age groups, but is especially common among young and middle-aged adults, often interfering with daily activities, work, and overall quality of life [2,3]. Prevalence and burden of migraine may vary across regions due to demographic, environmental, and lifestyle differences [2]. In addition to the characteristic headache, many individuals experience symptoms such as nausea, sensitivity to light or sound, and difficulty concentrating, which can further limit daily functioning [3]. From a clinical perspective, migraine is considered a complex neurovascular disorder involving altered neuronal excitability and dysregulation of pain-processing pathways, which may explain its variability across individuals [4].

Although pharmacological treatments are widely used, they do not always provide consistent relief. Many patients continue to experience frequent or severe episodes, which has led to growing interest in factors that may influence migraine beyond medication alone. In this context, lifestyle-related factors have received increasing attention, particularly because they are potentially modifiable and may contribute to both the development and management of migraine [5,6,7]. These factors are often interrelated and may act through shared mechanisms such as changes in metabolic balance, sleep regulation, or stress response [8].

Diet is one of the most commonly discussed factors. Some individuals report that certain foods or eating habits, such as skipping meals or consuming processed foods, can trigger migraine episodes. Proposed mechanisms include fluctuations in blood glucose levels, dehydration, and sensitivity to specific food components such as caffeine or additives [5,6]. At the same time, these relationships are not always consistent. What acts as a trigger for one person may have little or no effect on another, suggesting that diet may play a role, but not in a uniform way [9,10]. This makes it difficult to define clear dietary patterns associated with migraine.

Physical activity is another factor with mixed findings. Regular, moderate exercise is generally associated with improved overall health and may contribute to reduced stress levels, which could be beneficial for migraine [11]. However, in some cases, especially when the activity is intense or irregular, it has also been reported as a potential trigger [12]. This variability suggests that the effect of physical activity may depend on its intensity, consistency, and individual tolerance [13].

Sleep and stress are also closely linked to migraine. Irregular sleep, poor sleep quality, or changes in sleep duration have been associated with migraine occurrence [14]. Similarly, stress is one of the most frequently reported triggers, although its role is complex and not always easy to measure. It may operate in both directions, as migraine itself can also contribute to increased stress levels [15]. Stress-related physiological changes, including alterations in cortisol levels and autonomic nervous system activity, may contribute to migraine susceptibility. At the same time, the relationship is likely bidirectional, as recurrent migraine episodes can also increase perceived stress. Since these factors often interact, their combined effect may be more relevant than considering each factor separately [15,16].

While many studies have explored these variables separately, fewer have examined them together within the same population. Most available research tends to focus on individual factors, which may not fully reflect how these influences coexist and interact in everyday life. A more integrated approach may therefore provide a clearer understanding of how lifestyle patterns relate to migraine characteristics [17]. Assessing physical activity, dietary habits, sleep, and stress simultaneously may provide a more integrated view of how everyday behaviors relate to migraine patterns.

Therefore, this study aims to examine the relationship between physical activity, dietary habits, and selected lifestyle factors, including sleep and stress, with the frequency and intensity of migraine. In addition, it aims to identify modifiable lifestyle factors that may contribute to improved management of migraine. It was hypothesized that selected lifestyle factors, including physical activity, dietary habits, sleep, and perceived stress, would be associated with migraine frequency and intensity.

## 2. Materials and Methods

### 2.1. Study Design and Data Collection Site

This study was conducted as a cross-sectional survey aimed at exploring how physical activity, dietary habits, and selected lifestyle factors relate to migraine characteristics in adults. Data were collected through an online questionnaire distributed within an online migraine-focused community. The study was conducted from 1 January to 28 February 2026 and was reported in accordance with the Strengthening the Reporting of Observational Studies in Epidemiology (STROBE) guidelines [18].

### 2.2. Participant Recruitment and Sample Size Calculation

A total of 300 adults participated in the study. Participants were recruited through an online migraine-focused community, where individuals were invited to take part voluntarily. A formal sample size calculation was not performed, as the study was exploratory and based on feasibility.

Participation was voluntary, and all responses were collected anonymously, with no personal identifying information recorded. By completing the questionnaire, participants were considered to have provided informed consent.

### 2.3. Inclusion and Exclusion Criteria

To be eligible, participants had to be at least 18 years old and report a history of migraine, either self-reported or previously diagnosed by a healthcare professional. Participants with self-reported migraine were included based on self-identification of typical migraine features. However, formal diagnostic criteria, such as the International Classification of Headache Disorders (ICHD-3), were not applied [19]. This approach may have introduced some heterogeneity in the study population. No additional exclusion criteria were predefined beyond failure to meet the inclusion criteria or incomplete questionnaire responses.

### 2.4. Data Collection Instrument

Data were collected using a structured, self-administered questionnaire developed specifically for this study. The questionnaire was developed based on previously published instruments assessing migraine and lifestyle-related factors, including diet, sleep, and perceived stress, and was reviewed for clarity before distribution [5,6,9,14,16]. The selection of items was guided by the study objectives and existing literature. The questionnaire included sections on demographic characteristics, migraine features, and lifestyle factors, medication use, and dietary supplements.

Demographic data included age, gender, weight, and height, from which body mass index (BMI) was calculated. Migraine-related variables included duration of the condition, frequency of migraine episodes in the past three months, duration of attacks, and pain intensity. Migraine intensity was assessed using a numerical rating scale from 0 to 10. Migraine frequency and duration were recorded using predefined categories based on the questionnaire responses.

Lifestyle-related variables included physical activity, sleep duration, perceived stress, and dietary habits. Physical activity was assessed as the number of days per week with at least 30 min of activity. Sleep duration was recorded in categories based on average daily sleep. Perceived stress was self-rated on a scale from 0 to 10. Dietary habits were assessed through questions on meal regularity, skipping meals, daily water intake, fast food consumption, caffeine intake, alcohol use, and potential food triggers. Participants were also asked whether they noticed an increase in migraine episodes when skipping meals.

Additional variables included medication use (type and frequency), use of dietary supplements, and migraine characteristics such as aura and menstrual-related migraine.

### 2.5. Statistical Analysis

Data were analyzed using SPSS Statistics for Windows, version 27. Descriptive statistics were used to summarize the characteristics of the study population. Continuous variables were presented as mean values with standard deviations (SD), while categorical variables were reported as frequencies and percentages. Data were presented as mean ± SD for consistency with previous literature, although some variables were ordinal.

Given that several variables were ordinal in nature, including migraine frequency, duration of episodes, sleep duration, and physical activity, Spearman correlation analysis was used to examine correlations between migraine characteristics (frequency, intensity, and duration) and lifestyle factors such as physical activity, dietary habits, sleep, and stress.

Multiple linear regression analyses were conducted to identify factors associated with migraine intensity. The models included key lifestyle variables (physical activity, fast food consumption, sleep duration, caffeine intake, alcohol use, and perceived stress), along with demographic variables (age, gender, and BMI) and clinically relevant factors such as aura and menstrual-related migraine. Variables were selected based on clinical relevance and previous literature [20].

Group differences in migraine intensity and frequency were assessed using independent samples *t*-tests and one-way analysis of variance (ANOVA), particularly in relation to categorical variables such as aura, menstrual-related migraine, and medication use. Association tests such as chi-square or Fisher’s exact test were not included, as the study primarily focused on relationships assessed through correlation and regression methods. Where appropriate, post hoc comparisons were considered to further explore significant differences between groups.

A *p*-value of less than 0.05 was considered statistically significant in all analyses. Subgroup analyses (e.g., based on physician-diagnosed versus self-reported migraine) were not performed due to the exploratory nature of the study. Before interpreting the regression models, assumptions were checked by examining normality of residuals, linearity, homoscedasticity, and multicollinearity, including inspection of residual plots and variance inflation factors.

## 3. Results

**Figure 1:**
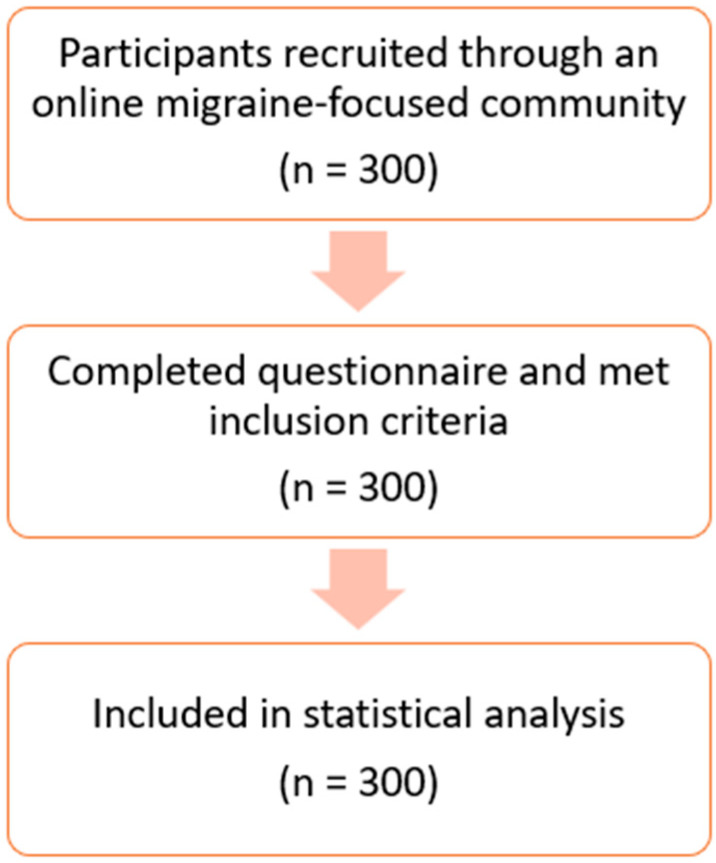
Flow diagram of participant recruitment and inclusion in the study.

A total of 300 participants were included in the analysis (Table 1). The majority of participants were female (88%). The mean age was 35.58 years (SD = 11.66), and the average BMI was 25.52 kg/m^2^ (SD = 3.05). Over the past three months, participants reported a mean migraine frequency score of 2.94 (SD = 1.23), while the mean migraine intensity was 7.44 (SD = 1.72), suggesting that many participants experienced relatively high levels of pain. The average episode duration score was 2.14 (SD = 1.10). Regarding lifestyle factors, the mean stress level was 6.74 (SD = 2.41) on a 0–10 scale indicating a generally elevated level of perceived stress in the sample. Physical activity levels were relatively low, with participants reporting an average of 1.05 days per week (SD = 0.90) of at least 30 min of activity. Dietary habits showed a mean fast food consumption score of 1.32 (SD = 0.95), while the mean sleep duration score was 2.16 (SD = 0.86). Alcohol consumption was reported with a mean score of 1.55 (SD = 0.57). The participant recruitment and inclusion process is shown in Figure 1.

Participants were also asked to identify foods they perceived as potential migraine triggers (Table 2). The most frequently reported triggers were alcohol (n = 53; 18%), sweets (n = 50; 17%), energy drinks (n = 46; 15%), and fast food (n = 44; 15%). Less commonly reported triggers included processed meat (n = 26; 9%), cheese (n = 24; 8%), citrus fruits (n = 13; 4%), and coffee (n = 10; 3%).

Migraine intensity was positively correlated with migraine frequency (rs = 0.31, *p* < 0.001) and episode duration (rs = 0.48, *p* < 0.001), indicating that more frequent and longer attacks were associated with higher pain intensity (Table 3). Smaller but statistically significant correlations were also observed with sleep duration (rs = 0.16, *p* < 0.05) and perceived stress (rs = 0.17, *p* < 0.05). Migraine frequency was positively associated with episode duration (rs = 0.40, *p* < 0.001), fast food consumption (rs = 0.23, *p* < 0.01), and BMI (rs = 0.25, *p* < 0.01), suggesting a possible link between dietary habits, body weight, and migraine occurrence. Physical activity was not significantly correlated with migraine frequency or intensity, but showed a weak positive correlation with perceived stress (rs = 0.19, *p* < 0.01).

In the multiple regression analysis, migraine frequency (β = 0.17, *p* = 0.005), perceived stress (β = 0.23, *p* = 0.006), and aura (β = −0.19, *p* = 0.033) emerged as significant predictors of migraine intensity (Table 4). Higher migraine frequency and higher perceived stress were associated with increased pain intensity, while the presence of aura showed a negative association with intensity in the adjusted model. In contrast, physical activity, fast food consumption, sleep duration, and menstrual migraine were not significantly associated with migraine intensity in the model. Overall, the model explained 14% of the variance in migraine intensity (R^2^ = 0.14, adjusted R^2^ = 0.12), and was statistically significant (F = 2.92, *p* = 0.007).

When examining group differences, participants who reported experiencing aura had higher migraine intensity (M = 7.84, SD = 1.70) compared to those without aura (M = 7.00, SD = 1.74) and those who were unsure (M = 7.00, SD = 1.57) (Table 5). This difference was statistically significant (F = 4.35, *p* = 0.015). Similarly, for menstrual migraine, participants who reported worsening symptoms around the menstrual cycle showed higher pain intensity (M = 7.56, SD = 1.68) compared to the other groups. This difference was statistically significant (F = 3.004, *p* = 0.032).

A comparison based on medication use showed that participants who did not use medication reported higher migraine intensity (M = 7.55, SD = 1.80) compared to those who used medication (M = 6.73, SD = 1.65), and this difference was statistically significant (*p* = 0.045) (Table 6). Regarding the type of medication, analgesics were the most commonly used (n = 118; 54%), followed by preventive therapies (n = 37; 17%) and triptans (n = 31; 14%). Other medications were reported by a smaller proportion of participants. In terms of supplement use, magnesium (n = 97; 32%) and vitamin D (n = 66; 22%) were the most frequently reported, followed by vitamin B complex (n = 55; 18%) and herbal preparations (n = 38; 13%).

## 4. Discussion

This study examined the relationship between key lifestyle factors, including physical activity, dietary habits, sleep, and perceived stress, and migraine characteristics in adults. The findings showed that migraine intensity was mainly associated with migraine frequency, episode duration, and perceived stress, while migraine frequency was also related to dietary factors and BMI. In contrast, physical activity did not show a direct association with migraine intensity or frequency.

The relationship between migraine intensity, frequency, and episode duration observed in this study is consistent with previous findings, but it also highlights how these dimensions of migraine tend to reinforce each other [2,6,21]. Individuals who experience more frequent migraine episodes are likely to have a greater cumulative burden of attacks, which may be associated with both longer duration and increased perceived intensity of pain over time. In addition, repeated or prolonged attacks may be linked to increased sensitization of central pain pathways, resulting in reduced pain tolerance and more severe subsequent episodes [22]. Similar patterns have been described in earlier studies, where migraine frequency and duration are often used as indicators of overall disease burden and severity [23,24].

Perceived stress was one of the factors most consistently associated with migraine intensity in this study, which is in line with previous research identifying stress as a common trigger for migraine attacks [14,16,25,26]. However, this relationship is unlikely to be one-directional. On one side, higher stress levels may be associated with a greater likelihood of migraine episodes, while on the other, frequent or severe migraines can, in turn, contribute to elevated perceived stress over time. This bidirectional relationship suggests a reinforcing cycle, where stress and migraine interact rather than act as independent factors. At a physiological level, stress-related changes, including alterations in cortisol regulation and autonomic nervous system activity, may contribute to increased migraine susceptibility [8,25,26]. In addition, central sensitization and dysregulation of stress-response pathways have been proposed as mechanisms linking chronic stress with increased pain perception [8].

Dietary factors in this study showed some relationship with migraine frequency, especially in relation to fast food consumption. Participants also reported alcohol, sweets, and energy drinks as common triggers. These findings are consistent with previous studies in which specific foods and dietary habits are frequently reported as potential migraine triggers [4,7,9,27,28,29,30]. However, the relationship between diet and migraine does not appear to be consistent across individuals. While some participants are able to clearly identify specific food-related triggers, others do not report any recognizable pattern. This variability suggests that dietary influences on migraine may be highly individual and may be influenced by factors such as eating habits, sensitivity to certain ingredients, and overall lifestyle. Several mechanisms have been proposed to explain this relationship, including fluctuations in blood glucose levels, dehydration, and the effects of bioactive compounds such as caffeine and food additives [10]. In addition, irregular eating patterns, particularly skipping meals, have been associated with an increased likelihood of migraine episodes [31,32]. Taken together, these findings suggest that overall dietary behavior may be more relevant than individual foods alone when considering the role of diet in migraine.

Physical activity was not significantly associated with migraine intensity or frequency in this study. This finding differs from some studies reporting a beneficial association of regular physical activity on migraine outcomes, including reductions in frequency and intensity [10,33]. However, the literature remains inconsistent, with several studies showing weak or non-significant associations, and in some cases identifying physical activity as a potential trigger, especially when it is intense or irregular [11,12,13]. In this study, physical activity showed a weak association with perceived stress, suggesting a possible indirect role. It is possible that physical activity influences migraine through mechanisms such as stress modulation rather than having a clear and direct effect on migraine frequency or intensity [34]. Variations in intensity, duration, and regularity of activity may further contribute to these inconsistent findings.

Sleep showed a small but significant association with migraine intensity in this study. This finding should be interpreted cautiously, as sleep duration was assessed in categories and may not reflect sleep quality. Although the effect was modest, it is consistent with previous research linking sleep disturbances and changes in sleep patterns with migraine [12,33,35]. Sleep is also closely connected to other lifestyle factors, particularly stress and daily routines, which makes it difficult to assess its role in isolation. Variations in sleep timing and quality, even when not fully captured by duration alone, may be related to how migraine is experienced, especially in individuals who are more sensitive to potential triggers.

BMI was also associated with migraine frequency, although the strength of the relationship was modest. This finding is consistent with studies suggesting that higher body weight may be linked with more frequent or more severe migraine episodes [36,37,38,39]. The underlying mechanisms are not fully understood, but proposed explanations include low-grade systemic inflammation, metabolic dysregulation, and shared lifestyle factors [36]. These mechanisms may interact rather than act independently, particularly in relation to diet and physical activity. Overall, these findings suggest that BMI may be related to migraine, but it is likely one component within a broader and more complex set of influencing factors. The observed association was modest and should be interpreted cautiously, particularly in the context of other contributing factors.

One of the more notable findings of this study is the higher migraine intensity observed among participants who reported aura. This is consistent with previous studies suggesting that migraine with aura may be associated with a more severe clinical presentation [23,24]. This difference may reflect underlying neurobiological distinctions, as migraine with aura is often considered a distinct subtype characterized by more complex neurological involvement [4]. As a result, symptoms may be more pronounced and the overall experience of migraine more severe in this group. Interestingly, while participants with aura showed higher migraine intensity in group comparisons, the regression analysis indicated a negative association after adjustment for other variables. This may reflect the influence of confounding factors, suggesting that the relationship between aura and migraine intensity is more complex when considered alongside other clinical and lifestyle variables.

A similar, although less pronounced, pattern was observed in relation to menstrual migraine. Participants who reported worsening symptoms around the menstrual cycle also showed slightly higher intensity levels. This finding is consistent with previous research linking hormonal fluctuations, particularly changes in estrogen levels, to migraine occurrence [12]. However, the magnitude of the difference was modest, suggesting that hormonal factors may contribute to migraine intensity, but are unlikely to act independently. This finding should also be considered in the context of the predominantly female sample, which may have influenced the observed pattern.

Participants who reported using medication had lower migraine intensity compared to those who did not, which is consistent with the expected role of both acute and preventive treatments in reducing pain severity and improving symptom control [23]. This difference may reflect more effective or timely management of migraine episodes, rather than medication use alone. However, medication use for migraine is not uniform and may be influenced by factors such as access to treatment, timing of administration, and adherence. Earlier use of appropriate medication, in particular, has been associated with improved pain outcomes in previous studies [40].

Regarding the types of medication and supplements, most participants reported using common options such as analgesics, while preventive therapies and triptans were used less frequently and more selectively. This pattern is consistent with clinical practice, where analgesics are generally more accessible and widely used. Magnesium and vitamin D were also commonly reported supplements, although evidence regarding their effectiveness in migraine remains mixed [13,28].

This study provides additional insight into how multiple lifestyle factors relate to migraine characteristics in a real-world population. From a clinical perspective, these findings suggest that management of migraine may benefit from a broader focus on modifiable lifestyle factors, particularly stress and dietary habits. While individual responses may vary, considering these factors together may support more personalized approaches to migraine management.

### Strengths and Limitations

This study provides an integrated assessment of migraine by examining physical activity, dietary habits, sleep, and stress within the same population. By considering multiple lifestyle factors simultaneously, it offers a more comprehensive perspective on how these variables relate to migraine characteristics. To our knowledge, few studies in this setting have explored these factors together, which adds relevant context to the existing literature. In addition, the relatively large sample size strengthens the reliability of the observed patterns. The use of a single data collection instrument across all variables ensured consistency in measurement, allowing for a more coherent assessment of these relationships. The study also reflects real-world conditions, capturing self-reported experiences of individuals outside of controlled clinical settings.

However, several limitations should be considered when interpreting the findings. The cross-sectional design does not allow conclusions about causality, and the use of self-reported data may introduce recall and reporting bias. Participants were recruited through an online migraine-focused community, which may limit the generalizability of the results to the broader population. The predominance of female participants may have influenced certain findings, particularly those related to menstrual migraine, and may further limit generalizability. In addition, some variables, such as sleep duration, were assessed using categorical measures, which may not fully capture aspects such as sleep quality. The study did not include subgroup analyses based on physician-diagnosed versus self-identified migraine, and analyses stratified by sex were not performed, which may have limited the exploration of potential differences across subgroups and introduced additional heterogeneity in the study population. In addition, the questionnaire was not formally validated, and sociodemographic variables such as education level, marital status, and income were not collected, which may affect the reliability of the measurements and limit further interpretation of the findings.

## 5. Conclusions

This study suggests that migraine is influenced by multiple interacting lifestyle factors rather than a single determinant. Perceived stress showed the most consistent association with migraine intensity, while dietary factors and BMI were related to migraine frequency. In contrast, physical activity did not demonstrate a clear direct association with migraine outcomes in this sample.

These findings support a more individualized approach to understanding migraine, where lifestyle factors may contribute differently across individuals. Rather than focusing on isolated triggers, it may be more relevant to consider how these factors interact within daily routines. Future research, particularly longitudinal studies, is needed to better clarify these relationships and to determine whether modifying these factors can meaningfully influence migraine outcomes.

## Figures and Tables

**Table 1:** Descriptive statistics for the main variables (n = 300).

Variable	Mean (M)	SD
Age (years)	35.58	11.66
BMI (kg/m^2^)	25.52	3.05
Migraine frequency (past 3 months)	2.94	1.23
Migraine intensity (0–10)	7.44	1.72
Episode duration	2.14	1.10
Stress level (0–10)	6.74	2.41
Physical activity (days/week)	1.05	0.90
Fast food consumption (times/week)	1.32	0.95
Alcohol consumption	1.55	0.57
Sleep duration	2.16	0.86

**Table 2:** Dietary triggers of migraine among participants.

Food Trigger	n	%
Alcohol	53	18
Sweets	50	17
Energy drinks	46	15
Fast food	44	15
Chocolate	34	11
Processed meat	26	9
Cheese	24	8
Citrus fruits	13	4
Coffee	10	3

**Table 3:** Spearman correlation matrix among the main variables.

Variable	1	2	3	4	5	6	7	8
1. Migraine intensity	—							
2. Migraine frequency	0.31 ***	—						
3. Episode duration	0.48 ***	0.40 ***	—					
4. Physical activity	0.15	0.02	0.09	—				
5. Fast food	−0.02	0.23 **	−0.04	0.15	—			
6. Sleep	0.16 *	0.07	0.07	0.03	−0.13	—		
7. Stress	0.17 *	0.16	0.09	0.19 **	0.05	−0.02	—	
8. BMI	−0.16	0.25 **	−0.03	0.03	0.16	−0.15	0.02	—

Note: * *p* < 0.05, ** *p* < 0.01, *** *p* < 0.001.

**Table 4:** Multiple regression analysis with migraine pain intensity as outcome variable.

Predictor	B	SE	β	*p*
Migraine frequency	0.38	0.13	0.17	**0.005**
Physical activity	0.23	0.16	0.13	0.159
Fast food consumption	0.18	0.16	0.10	0.266
Sleep duration	0.02	0.17	0.01	0.90
Perceived stress	0.12	0.04	0.23	**0.006**
Aura	−0.4	0.19	−0.19	**0.033**
Menstrual migraine	0.08	0.22	0.03	0.714

**Note:** Significant effects are printed in bold. Dependent variable: migraine intensity. **Model summary:** R^2^ = 0.14, Adjusted R^2^ = 0.12, F = 2.92, *p* = 0.007.

**Table 5:** One-way ANOVA analysis of migraine intensity according to aura and menstrual migraine status.

Variable	Group	Mean	SD	F	*p*	η^2^
Aura	Yes	7.84	1.70	4.35	0.015	0.06
	No	7.00	1.74			
	Not sure	7.00	1.57			
Menstrual migraine	Yes	7.56	1.68	3.004	0.032	0.015
	No	7.44	2.03			
	Not sure	6.94	1.81			

**Table 6:** Medication use, types of medications, and supplements among participants with migraine.

Variable	Category	n (%)	Migraine Intensity (Mean ± SD)	*p*
Medication use	No medication	81 (27%)	7.55 ± 1.80	0.045
	Medication use	219 (73%)	6.73 ± 1.65	
Type of medication *	Analgesics (Paracetamol, Ibuprofen, etc.)	118 (54%)		
	Preventive therapy (Beta-blockers, Topiramate, etc.)	37 (17%)		
	Triptans	31 (14%)		
	Other (e.g., Avamigran, Nimesulid, etc.)	33 (15%)		
Supplements *	Magnesium	97 (32%)		
	Vitamin D	66 (22%)		
	Herbal preparations	38 (13%)		
	Vitamin B complex	55 (18%)		
	Omega-3	30 (10%)		
	Melatonin	14 (5%)		

“*” Category header.

## Data Availability

The data presented in this study are available from the corresponding authors on reasonable request.

## Abbreviations

The following abbreviations are used in this manuscript:

BMI	Body Mass Index
SD	Standard Deviation
ANOVA	Analysis of Variance
ICHD	International Classification of Headache Disorders
STROBE	Strengthening the Reporting of Observational Studies in Epidemiology
